# Initiating an antimicrobial stewardship program with limited resources

**DOI:** 10.1186/1753-6561-5-S6-O41

**Published:** 2011-06-29

**Authors:** JM Boyce, A Tauman, J Kozakiewicz, A Fisher, W Hung, T Halpert, A Karjoo

**Affiliations:** 1 Medicine, Hospital of Saint Raphael, New Haven, USA; 2 VHA, Southport, CT, USA; 3 Pharmacy, Hospital of Saint Raphael, New Haven, USA

## Introduction / objectives

Antimicrobial stewardship (AMS) is an important strategy for improving patient safety, but many hospitals have limited resources to devote to AMS. We describe formation of an AMS program in a 400-bed hospital with relatively limited resources.

## Methods

We developed an AMS program, headed by a pharmacist and infectious diseases (ID) physician. Drug utilization reviews identified an expensive, broad-spectrum agent (piperacillin-tazobactam [P-T]) for initial targeting. An AMS form for listing clinical data and recommendations was developed. A daily list of patients receiving P-T was used to identify target patients for intervention. The pharmacist and ID physician made rounds on wards, placed AMS forms in patient charts, and spoke to caregivers regarding recommendations. Intravenous to oral conversions were performed by pharmacists. After 6 months of AMS activity revealed cost savings, hospital administration approved a ½-time pharmacist position for AMS.

## Results

In the first complete year of the AMS program, the hospital spent $277,833 less on anti-infectives (11% reduction in cost/adjusted patient-day) than in the previous year. Of this amount, $172,865 less was spent specifically for broad-spectrum agents (22% reduction in cost/adjusted patient-day for broad-spectrum agents). P-T purchases decreased by 20%, levofloxacin by 10% and vancomycin by 19% compared to the previous year. 90% of recommendations were accepted by caregivers. The number of new, nosocomial *Clostridium difficile* diarrhea cases/10,000 patient-days decreased by 28% compared to the previous year.

## Conclusion

An AMS program implemented with relatively few resources resulted in cost savings to the hospital and improved patient care.

## Disclosure of interest

None declared.

